# Sports Cardiology as a Career Pathway: Guidance for Interested Cardiovascular Trainees

**DOI:** 10.1016/j.jacadv.2025.102305

**Published:** 2025-10-28

**Authors:** Eli M. Friedman, Aaron L. Baggish, Eugene H. Chung, Alfred Danielian, Elizabeth H. Dineen, J. Sawalla Guseh, Matthew W. Martinez, Caroline E. Murphy, Ankit B. Shah, Jonathan H. Kim, ACC Council on Sports and Exercise Cardiology

**Affiliations:** a Smidt Heart Institute, Cedars-Sinai, Los Angeles, California, USA; b Centre Hospitalier Universitaire Vaudois, Lausanne, Switzerland; c Cardiovascular Performance Program, Cardiovascular Division, Massachusetts General Hospital, MGH Heart and Vascular Institute, Boston, Massachusetts, USA; d Division of Sports Cardiology, Las Vegas Heart Associates, Las Vegas, Nevada, USA; e Department of Cardiovascular Medicine, Mayo Clinic in Florida, Jacksonville, Florida, USA; f Atlantic Health System, Morristown, New Jersey, USA; g U.S. Army Research Institute Research Institute of Environmental Medicine, Natick, Massachusetts, USA; h Sports and Performance Cardiology, Chevy Chase, Maryland, USA; i Division of Cardiology, Emory Clinical Cardiovascular Research Institute, Emory University School of Medicine, Atlanta, Georgia, USA

**Keywords:** sports, career, education, health system

**Figure:**
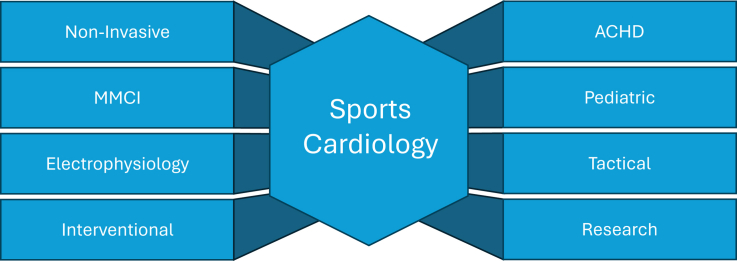

Sports cardiology has grown rapidly over the last decade. The intersection of cardiovascular disease (CVD) and high-intensity exercise is complex, requiring a thoughtful clinical approach.^1^ This specialty should be considered a necessity for tertiary care cardiovascular service lines and academic cardiology programs, given the increasing number of active patients with CVD and trainees interested in the field. As the role of the sports cardiologist becomes more defined and the demand for the specialty grows, it is critical to provide guidance to the next generation of cardiovascular specialists on how to develop careers in sports cardiology and institutions on how to build successful sports cardiology programs. The purpose of this viewpoint is to address career pathways in sports cardiology for trainees, discuss educational resources available, and provide rationale as to why a robust sports cardiology program is essential to a successful cardiology service line.

Presently, although there is no dedicated certification or required postgraduate fellowship for sports cardiology in the United States, there are many pathways available for trainees to develop the education and skills necessary to competently see competitive athletes and highly active people in clinical practice. There are currently a small number of sports cardiology fellowships in the United States. These programs are available after completion of a general cardiology fellowship and are sometimes combined with training in genetic cardiomyopathies. Althoughit is likely that the number of these training opportunities will increase, there are currently not enough programs available to meet the demand for qualified sports cardiologists in the United States. As such, it is imperative for trainees to seek sports cardiology mentors at their respective institutions and be aware of the resources that aid in building the knowledge base for sports cardiology practice. If no mentors are available locally, trainees are encouraged to look outward for guidance.

In 2017, a sports cardiology core curriculum document emphasized the skills necessary to become a practicing sports cardiologist.^1^ It is fundamental that a sports cardiologist is able to appropriately evaluate competitive athletes during a preparticipation exam, differentiate of exercise-induced cardiac remodeling from pathology, evaluate symptomatic athletes, and have comfort in participating in return-to-play discussions with athletes with CVD through shared decision-making. A sports cardiologist is required to have knowledge of the recent guidelines for competitive sports participation for athletes with cardiovascular abnormalities and have the appropriate tools available to risk stratify athletes with underlying cardiovascular risk and/or CVD.^2^ As the field looks to the future, we encourage sports cardiology education for all cardiology fellows with more in-depth pathways and training for those who wish to pursue careers in the field.

Additional opportunities exist within the American College of Cardiology’s (ACC) Sports and Exercise Cardiology section. The section represents a hub for any health care practitioner interested in sports cardiology and/or who takes care of athletic patients. Members gain insight into the latest science and education in sports cardiology through the official webpage and through ACC’s annual meetings, including the Scientific Sessions and the Care of the Athletic Heart. The ACC Sports Cardiology Section and Council have been responsible for shaping the modern landscape of the field through multiple high-level publications.^2^, ^3^, ^4^, ^5^ The section also provides opportunities for mentorship and research collaboration via connections to leaders and experts in the field. Through various mechanisms at the state and national levels, members of the section have led advocacy efforts emphasizing cardiac safety during athletic participation. The section also participates in grassroots community service with options for anyone to be involved. Those interested are encouraged to engage with the section and also their respective state ACC chapters.

Finally, engaging in science and scholarly activity is encouraged for all trainees interested in sports cardiology. Research specific to exercise physiology and sports cardiology has dramatically increased, although uncertainties in the field continue to exist, which provide ongoing opportunity for meaningful contribution. Many former, early career researchers interested in the science of sports cardiology now represent scientific leaders in the field based on their respective body of published scientific evidence.

There are currently many pathways for one to develop a career within sports cardiology (Figure 1). The classic sports cardiologist is traditionally a noninvasive clinical cardiologist well-skilled in general cardiology fundamentals combined with a thorough understanding of the unique cardiovascular features of an athlete. Critical tenets that require expertise include the accurate interpretation of athletic electrocardiograms and echocardiograms. Stress testing is a keystone of any cardiology practice. Stress testing athletes, however, should come with customized exercise protocols that generally extend beyond traditional protocols. In addition to studies such as stress echocardiography and nuclear imaging, sports cardiology programs often employ cardiopulmonary exercise testing to study the physiology of an athlete with symptoms or established CVD more intensely.

**Figure 1:**
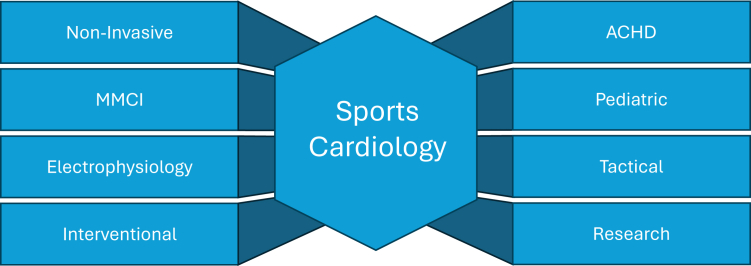
**Pathways to a Sports Cardiology Career** ACHD = adult congenital heart disease; MMCI = multimodality cardiac imaging.

Novel pathways have evolved that also facilitate a career focused on sports cardiology. Multimodality cardiovascular imaging (MMCI) is a critical tool used in the evaluation of athletic patients. Although a transthoracic echocardiogram is the most frequent study obtained, cardiac magnetic resonance imaging and coronary computed tomography angiography are frequently relied on in the diagnostic evaluation of athletes. In a high-volume sports cardiology practice, MMCI is used frequently and provides a career pathway for interested trainees. Obtaining certification in cardiac magnetic resonance imaging and coronary computed tomography angiography also allows the use of these skills in other patient populations. MMCI expertise is a primary clinical strategy that aids in the differentiation of exercise-induced cardiac remodeling from pathology. The American Society of Echocardiography provided the first clinical reference document for the use of MMCI in the clinical evaluation of young competitive athletes.^6^

Buoyed by the Heart Rhythm Society 2024 consensus statement on arrhythmias in athletes,^7^ the emergence of sports electrophysiology as a career pathway has evolved. Arrhythmias and arrhythmic conditions are commonly encountered in athletes that require expert electrophysiology involvement. Atrial fibrillation, for example, is frequently encountered in the care of masters-aged athletes. Wolff-Parkinson-White pattern is described as being one of the most common abnormalities found on electrocardiogram screening in athletes^8^ and comes with controversial clinical risk stratification and management considerations.^2^^,^^7^ In addition, both young and masters athletes may train competitively or at high intensities with defibrillators or pacemakers and require individualized programming of device parameters. Although arrhythmia management for athletes and nonathletes is often the same, care should be individualized, involve an electrophysiologist experienced in the care of athletes, and incorporate shared decision-making especially when discussing procedures.

Other burgeoning sports cardiology subspecialties are garnering traction as traditional cardiology subspecialists recognize unique issues athletes commonly present with that require nuanced clinical deliberation. Management of coronary artery disease in masters athletes comes with complex considerations compared to the general population. In particular, revascularization for stable coronary artery disease in masters athletes contrasts with guidelines applied to the general population. As such, sports interventional cardiology has recently emerged to handle these cases that require understanding of the differential risk present among highly active masters athletes. Tactical athletes (eg, military personnel, firefighters, and law enforcement) require expert oversight from experienced practitioners in the management of cardiovascular concerns. Given the specifications and strict requirements for those in tactical disciplines, it is important that sports cardiologists involved in the care of tactical athletes are available to maintain health and performance along with organizational standards.^9^ Athletes with congenital heart disease (CHD) and pediatric athletes also require expert care from sports cardiologists with expertise in these disciplines.^2^^,^^3^ Patients with CHD are living longer and can exercise vigorously, thus it is vital to understand the intersection between intense exercise and CHD to allow these patients to exercise safely.

Finally, pursuit of an academic research career is available in all fields in cardiology and sports cardiology is no exception. Advancements in clinical sports cardiology have accelerated over the last decade because of the emphasis on high-quality science and translational clinical research. A career in sports cardiology can combine the dedicated pursuit of extramural funding for protected research time in combination with a robust and fully dedicated sports cardiology clinical practice.

As the field of cardiology diversifies, sports cardiology should be included as an essential discipline in a successful cardiology service line. This success is evident in the care provided to athletes across the age spectrum and the contributions to other service lines that stem from the care of this group. Invariably other subspecialties within cardiology will be required to support athletes. A robust sports cardiology program benefits the entirety of the cardiovascular service line as athletic patients will often feed into the many other subspecialties of cardiology. Examples include the need for MMCI, subspecialty referral, and procedures which drive service and revenue for the division. Finally, as the complexities, nuances, and challenges associated with the cardiovascular care of elite athletes continue to increase, it is imperative that leagues, organizations, and sponsoring health care corporations recognize the critical need to have sports cardiology expertise available. This requires an appropriate compensation model for these expert providers for the unique, but essential care provided. Consideration of the institutional value and visibility that a sports cardiology program provides is imperative and may require compensation outside of traditional hospital/office-based models.

Although the field of sports cardiology is cemented as a valuable subspecialty in cardiology, there remains an unmet need for all patients who desire this care. It is critical that there is emphasis placed on ensuring sports cardiology education is included as part of traditional cardiovascular training curricula, establishing training pathways for fellows interested in sports cardiology, and ensuring trainees are aware of all resources available to build a sports cardiology-specific fund of knowledge. Cardiovascular service lines should continue to develop sports cardiology programs and recognize the support sports cardiology provides to patients and other subspecialties in the division. As the need for formal sports cardiology certification will likely arise in the future, advancing the educational initiatives in the field is a critical actionitem. There are numerous pathways to consider for a successful career in the field and all trainees and early career physicians should be aware of these options across all disciplines in cardiology. The future is bright and stakeholders should continue to engage through clinical, academic, and advocacy avenues to make the field accessible to all who desire it.

## Funding support and author disclosures

Dr Friedman has received compensation from the Women’s Tennis Association and Ultimate Fighting Championship’s Combat Sports Anti-Doping Program as a consultant. Dr Kim has received research funding from the 10.13039/100000002National Institutes of Health, 10.13039/100019115National Football League Players Association, and the Atlanta Track Club and compensation for his role as team cardiologist for the Atlanta Falcons, Atlanta Braves, Atlanta Hawks, and Atlanta United. Dr Baggish has received funding from the 10.13039/100000050National Institute of Health/National Heart, Lung, and Blood Institute, the 10.13039/100019115National Football League Players Association (NFLPA), and the 10.13039/100000968American Heart Association and receives compensation for his role as team cardiologist from the U.S. Olympic Committee/U.S. Olympic Training Centers, U.S. Soccer Federation, National Women’s Soccer League, and Lidl-Trek Cycling. Dr Martinez receives grant funding/consultancy compensation from the National Hockey League, National Basketball Players Association and Major League Soccer. Dr Shah has received compensation for his role as sports cardiology consultant from the U.S. Olympic and Paralympic Committee and MedStar Health; is on the Speakers Bureau for Bristol Myers Squibb; and is a consultant for Fourth Frontier. This article does not reflect the official policy of the Department of Defense or the U.S. Government. All other authors have reported that they have no relationships relevant to the contents of this paper to disclose.
